# Supplementary material for: Climate change is narrowing and shifting prescribed fire windows in western United States
Source: Commun Earth Environ. 2023 Oct 3;4(1):340. doi: 10.1038/s43247-023-00993-1 (PMC11041722; doi:10.1038/s43247-023-00993-1)
Supplement: Supplementary file 1 — Supplementary Material [file 43247_2023_993_MOESM1_ESM.docx]

Supplementary Information

For

**“Climate change is narrowing and shifting prescribed fire windows**

**in western United States”**

*Daniel L. Swain, John T. Abatzoglou, Crystal Kolden, Kristen Shive, Dmitri A. Kalashnikov, Deepti Singh, and Edward Smith*

**Figure S1.**

**
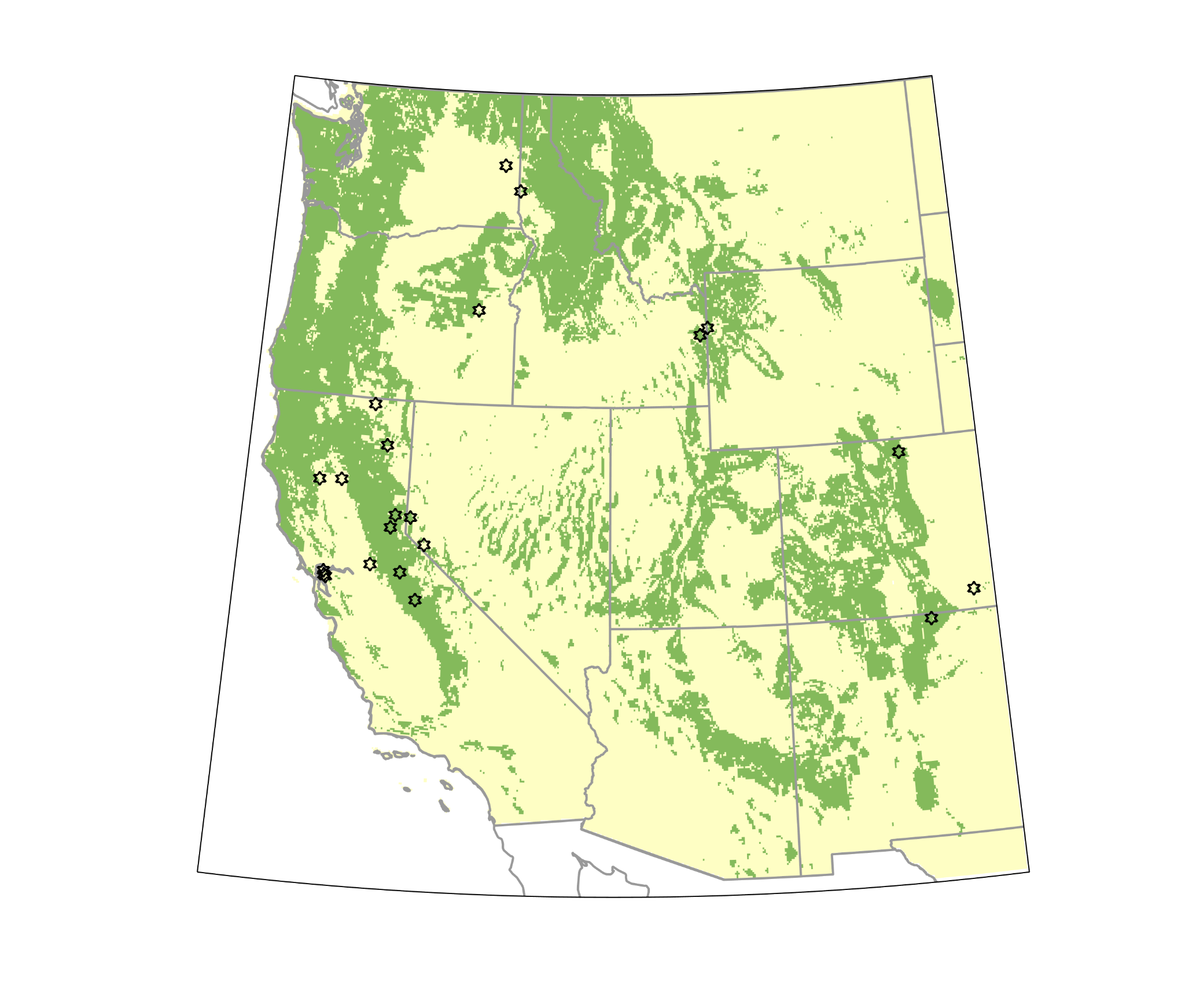
**

**Map depicting the Western United States study domain.** Regions in green shading represent portions of the domain considered to be primarily forest-covered for the purposes of this study; all other regions (i.e., “non-forest regions”) are shaded in yellow. Approximate locations of planned or implemented prescribed fires associated with the 22 burn plans used in this study are represented by open stars on the map.

**Figure S2.**

**
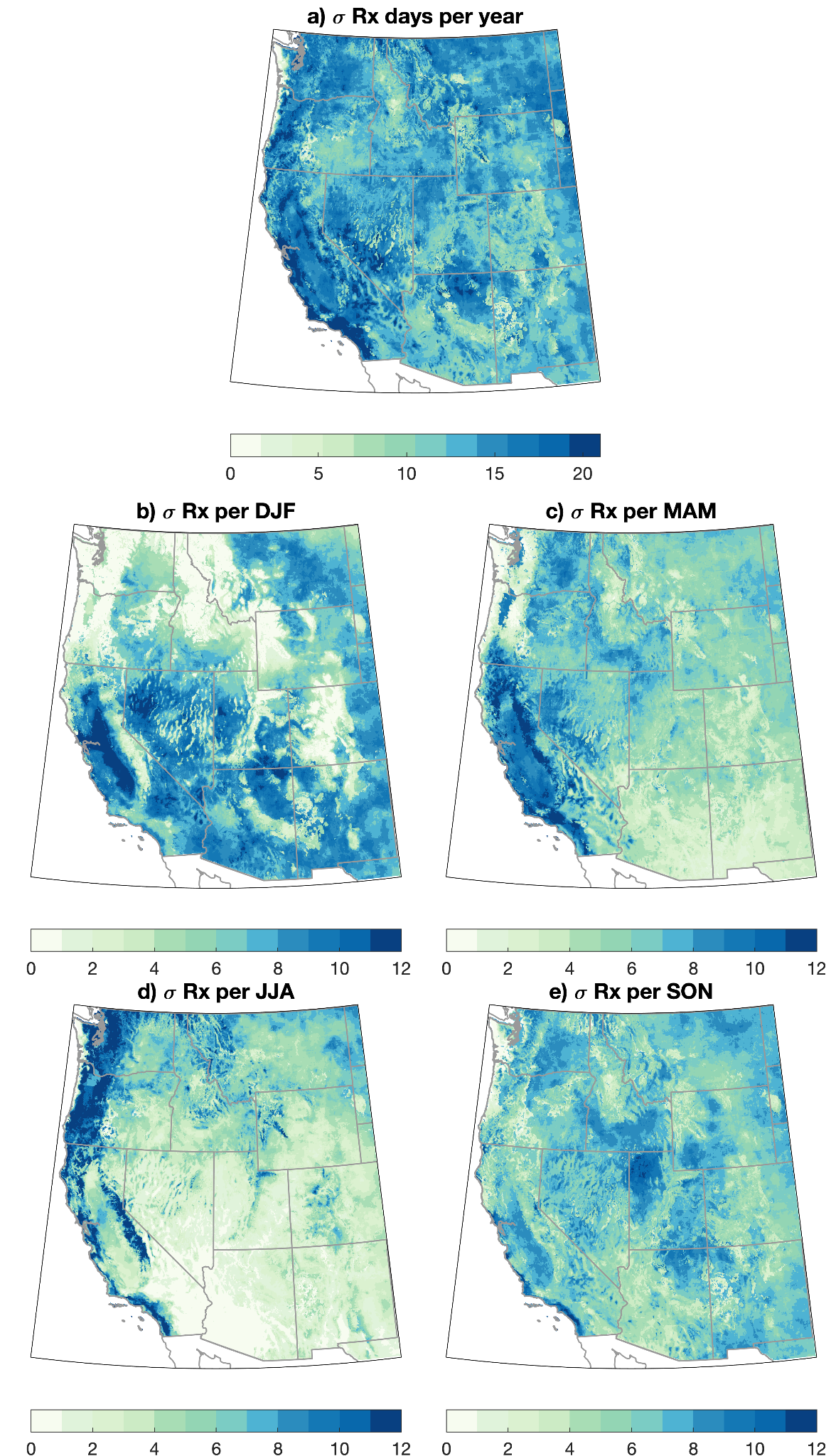
**

**Maps of observed RxDay variability across the WUS.** Maps depicting the standard deviation in the observed number of RxDays across the western United States (WUS) on an annual (a.) and seasonal (b.-e.) basis. Observed RxDays are calculated using meteorological data from the gridMET dataset over the years 1981-2020.

**Figure S3.**

**
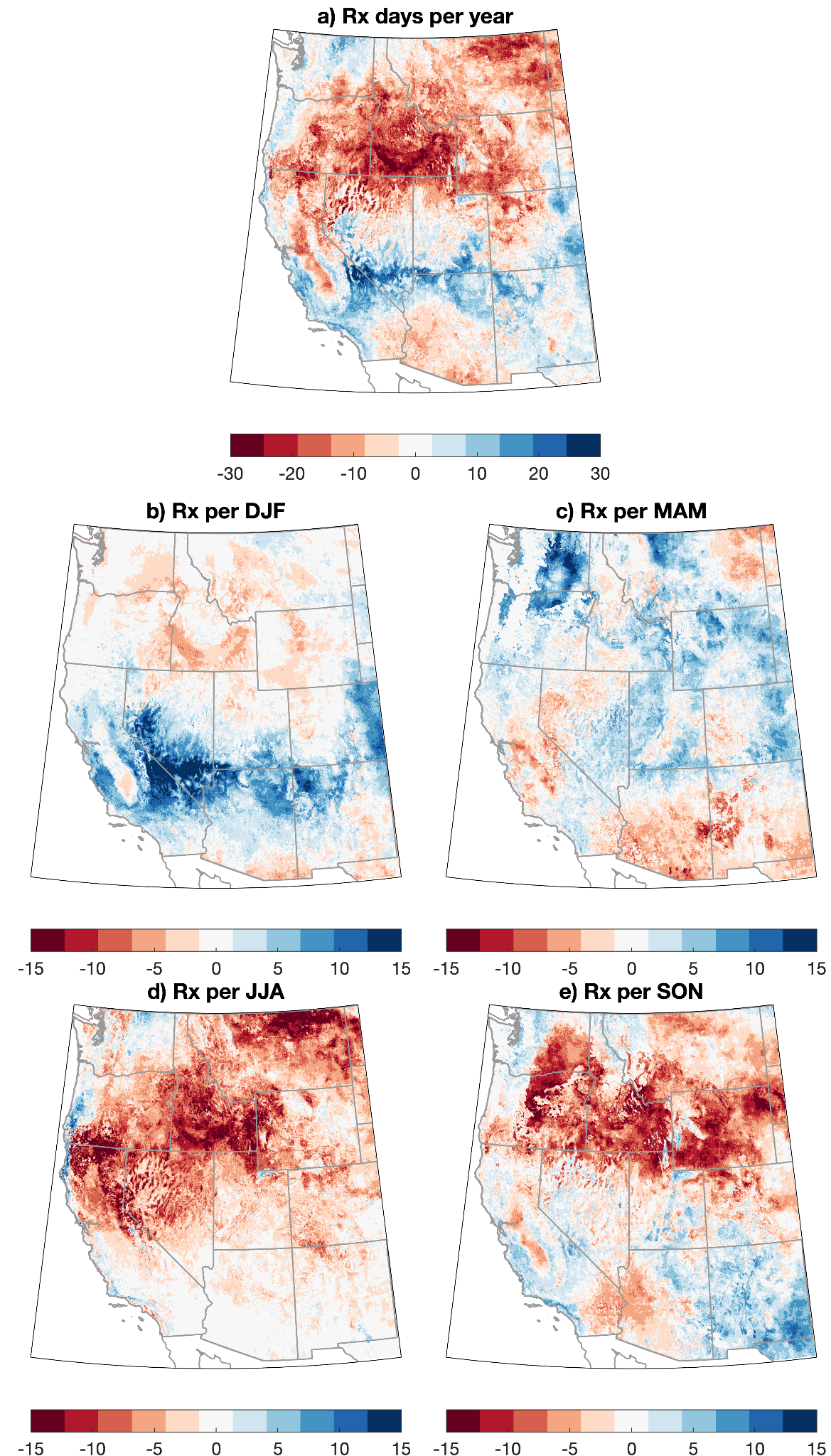
**

**Maps of projected trends in RxDays across the WUS, 1981-2020.** Maps depicting the projected change in the number of simulated historical RxDays across the western United States (WUS) on an annual (a.) and seasonal (b.-e.) basis. The trend in RxDays is calculated using a linear least squares regression, and is represented graphically by the accumulated change (in RxDays per year or season) over the full 40 year period using meteorological data from the downscaled CMIP5 climate model ensemble (MACA) dataset (1981-2020).

**Figure S4.**

**
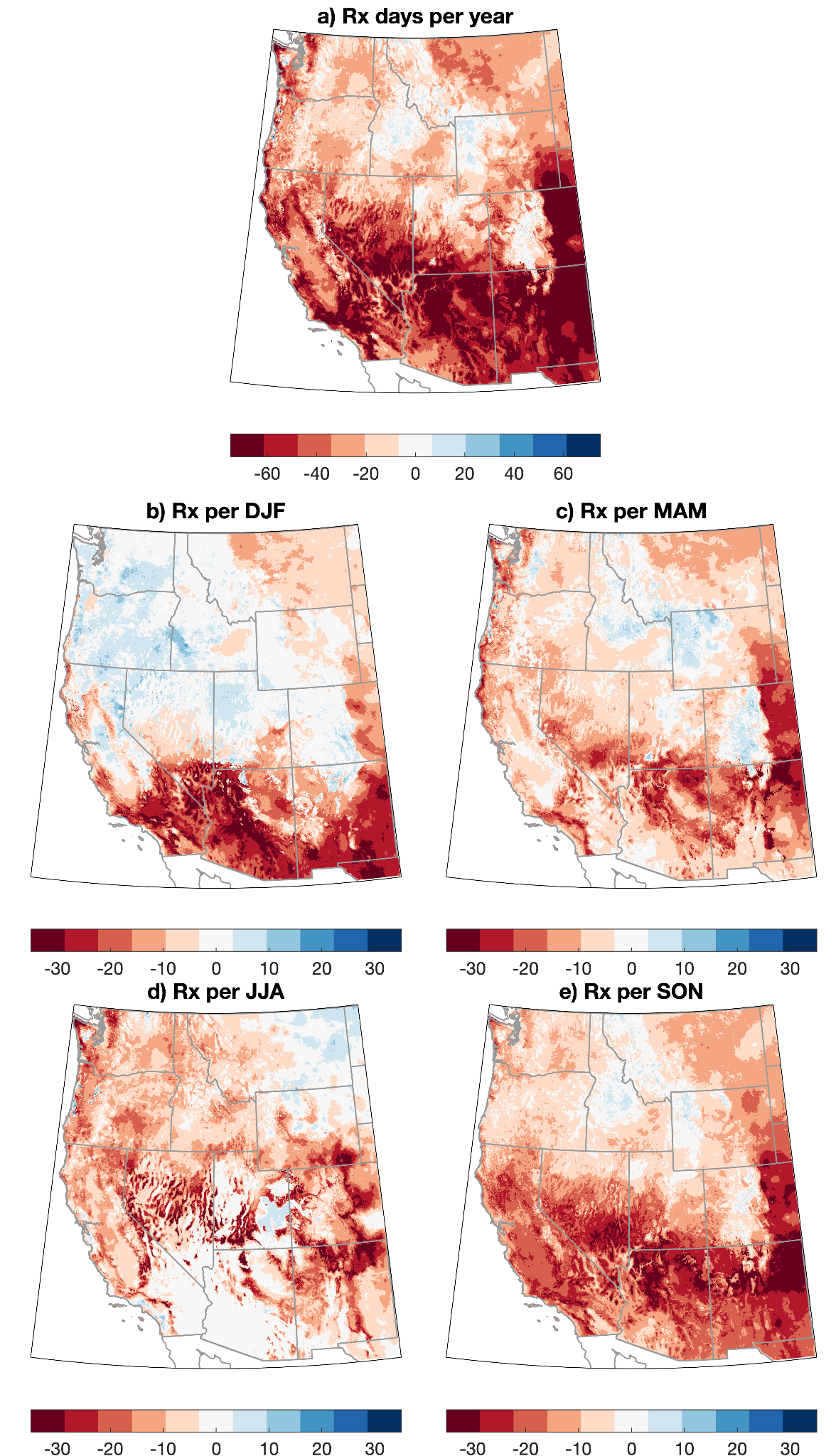
**

**Maps of observed trends in RxDays only considering meteorological criteria across the WUS, 1981-2020.** Maps depicting the change in the number of observed RxDays across the western United States (WUS) on an annual (a.) and seasonal (b.-e.) basis only using criteria for temperature, relative humidity, and wind speed. The trend in RxDays is calculated using a linear least squares regression, and is represented graphically by the accumulated change (in RxDays per year or season) over the full 40 year period using meteorological data from the GridMet dataset (1981-2020). Note that absolute units are not comparable to other RxDay plots as the exclusion of vegetation moisture criteria greatly increases the baseline number of RxDays.

**Figure S5.**


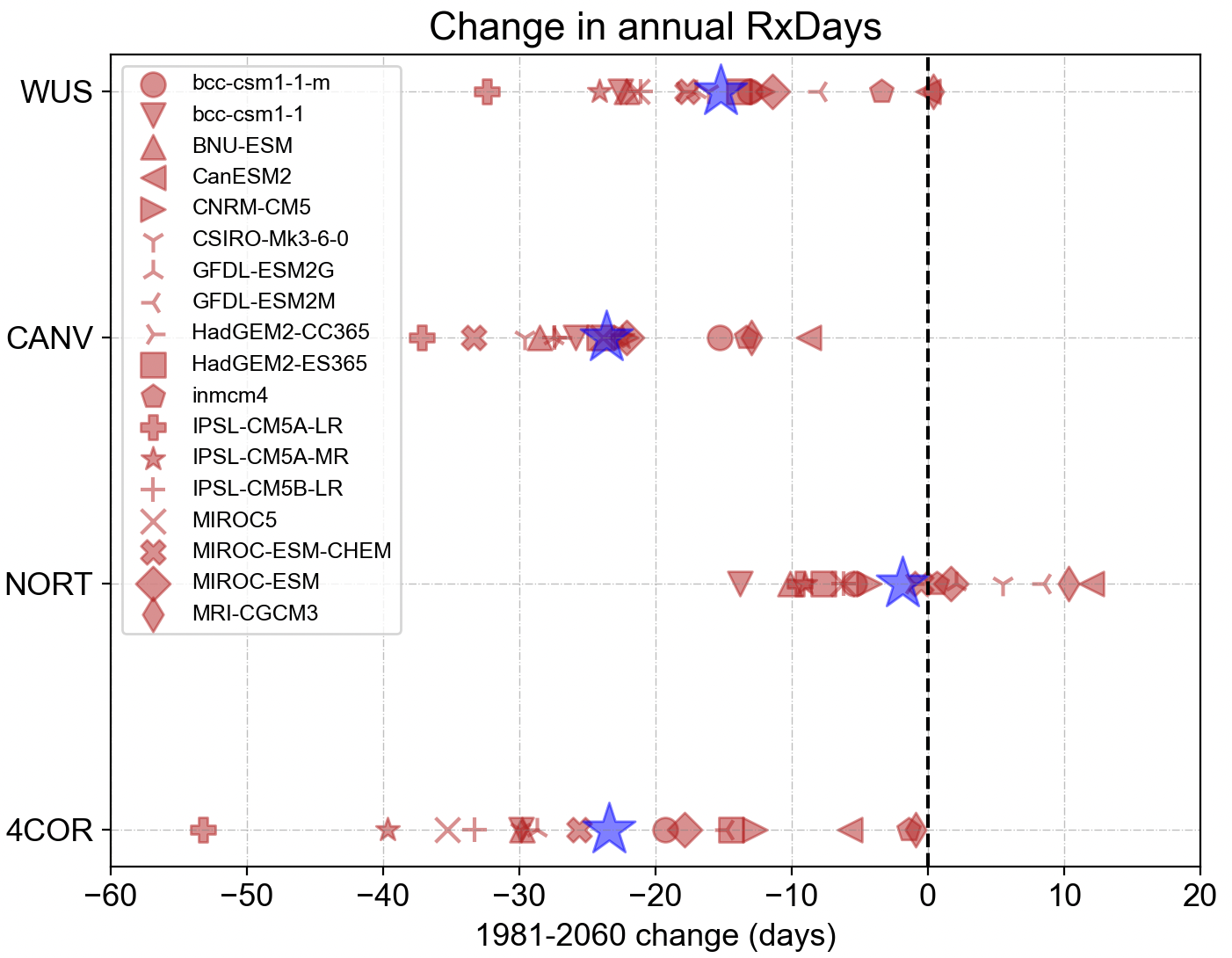


**Ensemble spread of projected trends in annual RxDays.** Dot plot depicting domain-averaged projected trends in cumulative annual RxDays days between 1981 and 2060 separately for each individual climate model (red shapes) used in this study for the full Western United States domain (WUS) and each subregion (CANV, NORT, and 4COR) (again using downscaled CMIP5 climate model data [MACA] and assuming a RCP4.5 “moderate warming” trajectory). Blue stars depict the ensemble mean trend for each subregion.

**Figure S6.**

**
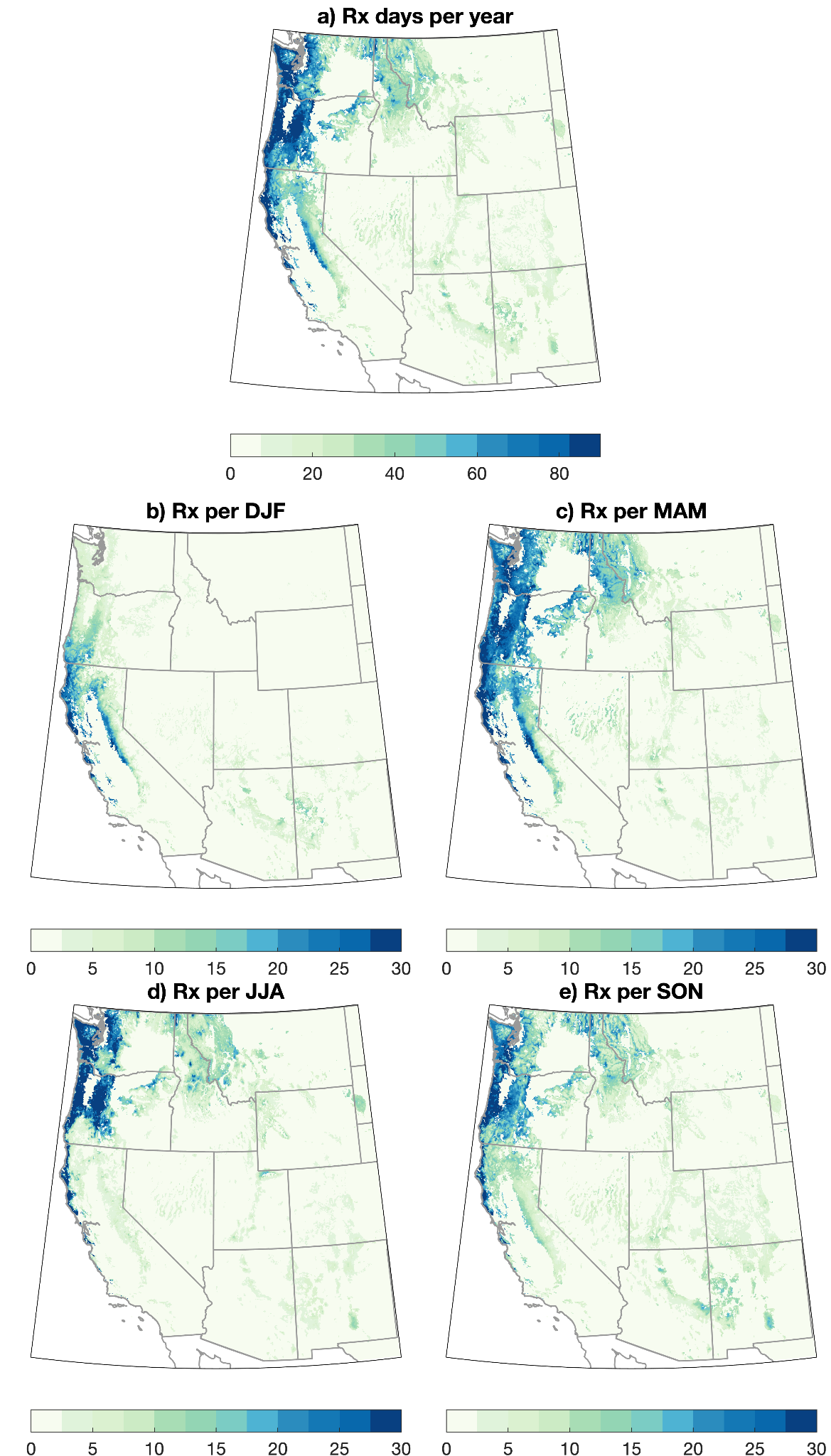
**

**Maps depicting importance of medium to large diameter fuel moistures in RxDay counts.** Maps depicting the difference in the number of RxDays across the western United States (WUS) on an annual (a.) and seasonal (b.-e.) basis depending on whether medium to large diameter fuel moisture (i.e., 100-hr and 1000-hr fuel moistures) are included as parameters in the RxDay definition. Note that all values are positive. Differences are calculated using observed meteorological data from the gridMET dataset over the years 1981-2020.

**Figure S7.**


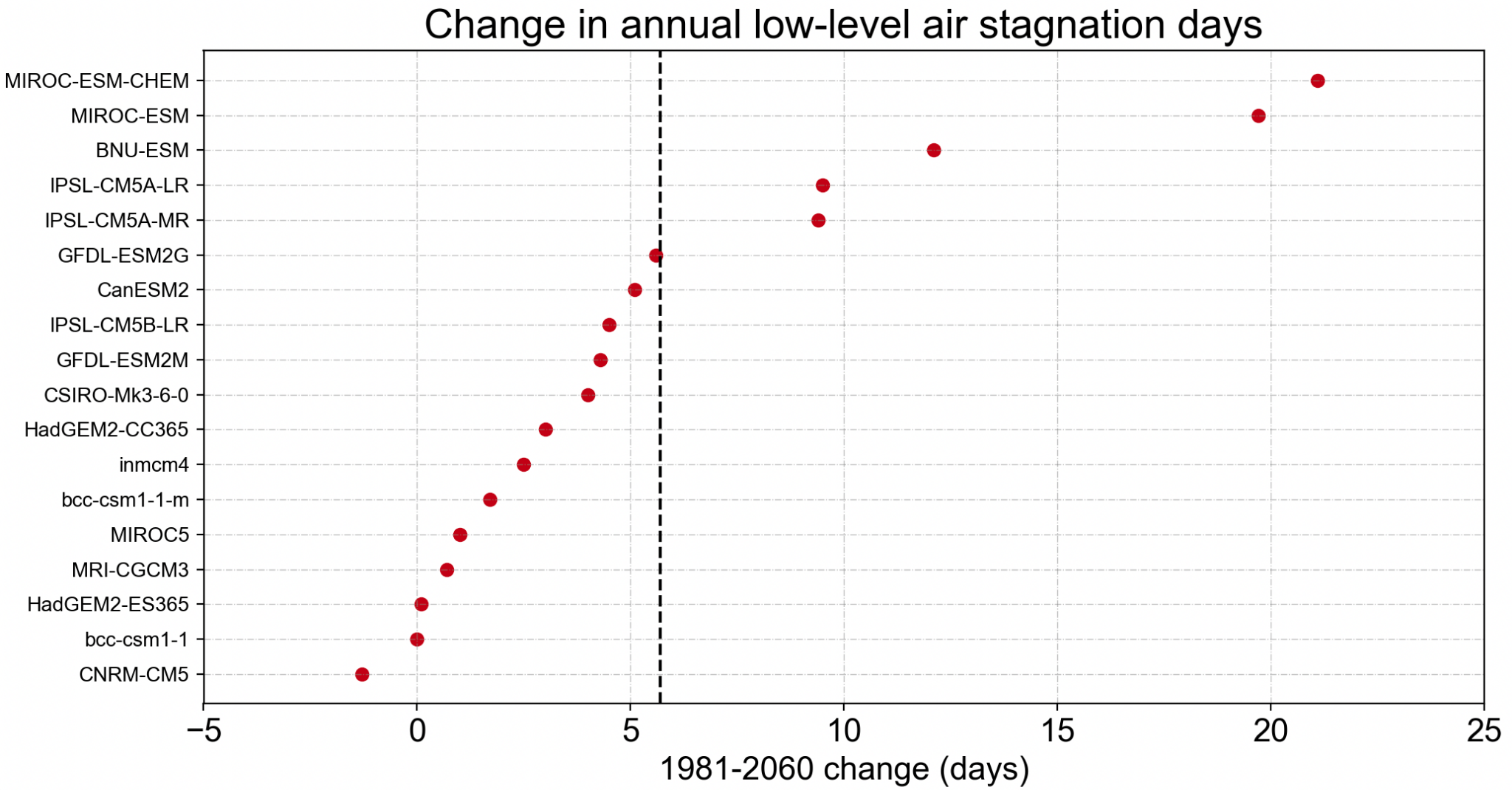


**Ensemble spread of projected trends in annual low-level air stagnation days, WUS.** Dot plot depicting domain-averaged projected trends in cumulative annual low-level air stagnation (LLS) days between 1981 and 2060 separately for each individual climate model used in this study (again using downscaled CMIP5 climate model data [MACA] and assuming a RCP4.5 “moderate warming” trajectory).

**Figure S8.**

**Maps depicting baseline RxDay sensitivity to different RxDay definitions.** Maps depicting the difference in the number of RxDays across the western United States (WUS) on an annual basis depending on which specific RxDay parameter ranges are used. **a)** Annual RxDays using the median environmental condition parameters from all available burn plans. **b)** Annual RxDays using the median environmental condition parameters only from the subset of burn plans conducted in primarily forested settings. **c)** Annual RxDays using the median environmental condition parameters only from the subset of burn plans conducted in primarily non-forested settings. All RxDays are calculated using observed meteorological data from the gridMET dataset over the years 1981-2020.
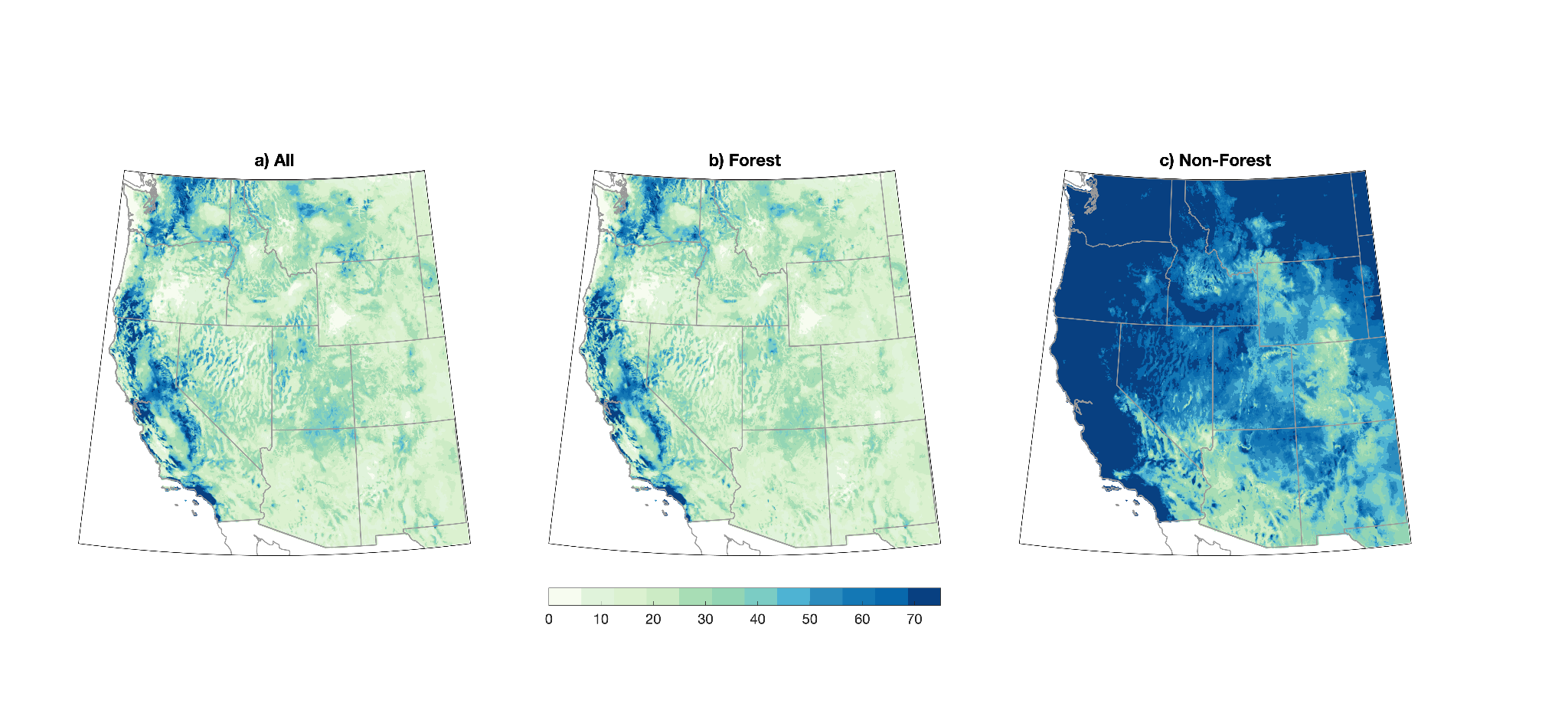


**Figure S9.**

**Maps depicting sensitivity of projected RxDay trends to different RxDay definitions.** Maps depicting the difference in protected trends in RxDays across the western United States (WUS) on an annual basis depending on which specific RxDay parameter ranges are used. **a)** Change in annual RxDays using the median environmental condition parameters from all available burn plans. **b)** Change in annual RxDays using the median environmental condition parameters only from the subset of burn plans conducted in primarily forested settings. **c)** Change in annual RxDays using the median environmental condition parameters only from the subset of burn plans conducted in primarily non-forested settings. All RxDays are calculated using observed meteorological data from the MACA dataset over the years (1981-2060).
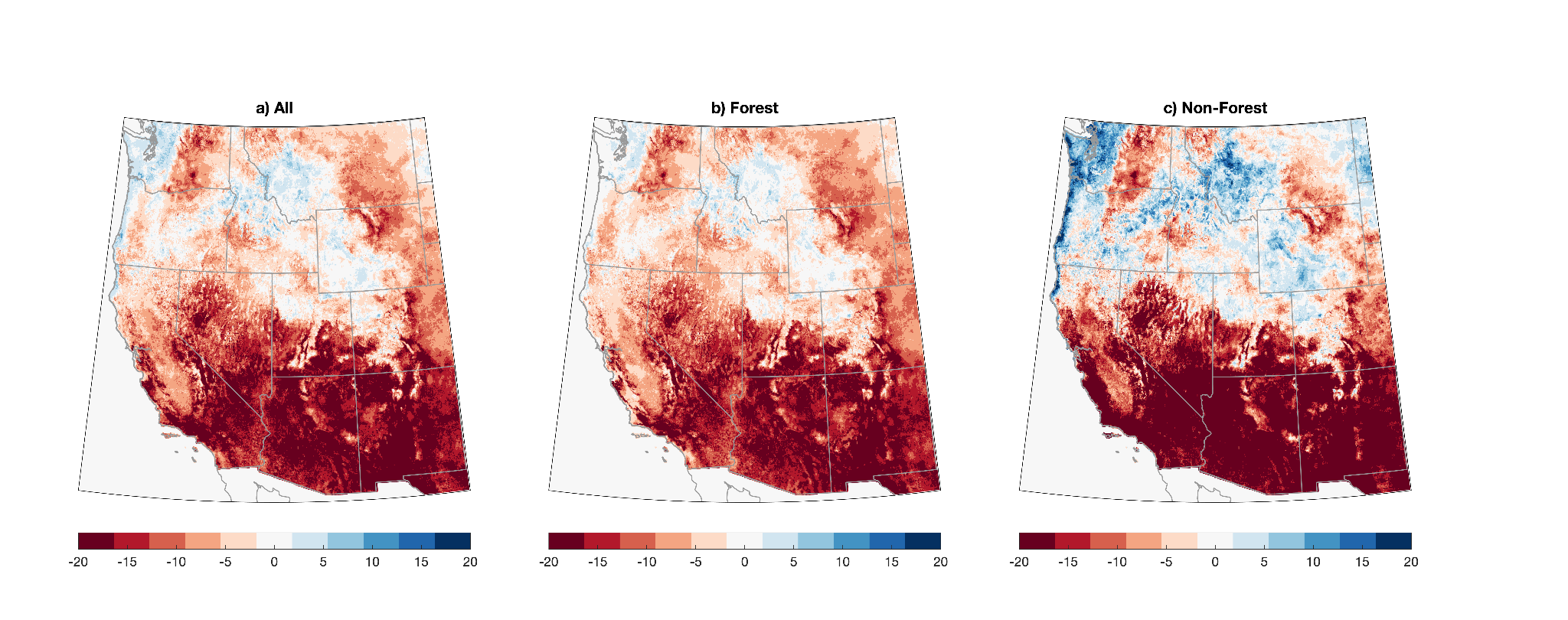


**Table ST1:** Comprehensive list of prescribed fire criteria used in this study. For each meteorological and fuel moisture metric we report the range of acceptable conditions to be within prescription. Ranges were taken as the median lower and medium upper bounds from existing burn plans. We report ranges for all plans, plans in forested environments, and plans in non-forested environments.

| **RxDay Constituent Variable** | **All Plans** | **Forest Plans** | **Non-Forest Plans** |
| --- | --- | --- | --- |
| Number of burn plans | 22 | 14 | 8 |
| 2m Air temperature (°C) | 5.8-29.4 | 7.2-28.9 | 4.4-29.4 |
| 2m Relative humidity (%) | 16-60 | 15-60 | 20-70 |
| Scaled 10m (mid-flame) wind speed (m/s) | 0-5.0 | 0-4.5 | 0-5.4 |
| Ignition component (units) | 20-65 | 20-75 | 21-60 |
| 1 hour fuel moisture (%) | 4-12 | 4-12 | 4-13 |
| 10 hour fuel moisture (%) | 6-14 | 6-14 | 6-15 |
| 100 hour fuel moisture (%) | 7.5-15 | 7.5-15 | – |
| 1000 hour fuel moisture (%) | 10-20 | 10-20 | – |

**Table ST2.** List of the 18 climate models from the Coupled Model Intercomparison Project, Phase 5 (CMIP5) used in the study along with the native resolution of the data. The first ensemble member for each model was used.

| **CMIP5 Climate Model** | **Resolution** |
| --- | --- |
| **Name** | **Latitude x Longitude** |
| bcc-csm1-1-m | 1.1215°x1.125° |
| bcc-csm1-1 | 2.7905°x2.8125° |
| BNU-ESM | 2.7905°x2.8125° |
| CanESM2 | 2.7905°x2.8125° |
| CNRM-CM5 | 1.4008°x1.4063° |
| CSIRO-Mk3-6-0 | 1.8652°x1.875° |
| GFDL-ESM2G | 2.0225°x2.5° |
| GFDL-ESM2M | 2.0225°x2.5° |
| HadGEM2-CC365 | 1.25°x1.875° |
| HadGEM2-ES365 | 1.25°x1.875° |
| inmcm4 | 1.5°x2° |
| IPSL-CM5A-LR | 1.8947°x3.75° |
| IPSL-CM5A-MR | 1.2676°x2.5° |
| IPSL-CM5B-LR | 1.8947°x3.75° |
| MIROC-ESM-CHEM | 2.7905°x2.8125° |
| MIROC-ESM | 2.7905°x2.8125° |
| MIROC5 | 1.4008°x1.4063° |
| MRI-CGCM3 | 1.1215°x1.125° |
